# Production and purification of homogenous recombinant human selenoproteins reveals a unique codon skipping event in *E. coli* and GPX4-specific affinity to bromosulfophthalein

**DOI:** 10.1016/j.redox.2021.102070

**Published:** 2021-07-17

**Authors:** Qing Cheng, Antonella Roveri, Giorgio Cozza, Luciana Bordin, Isabelle Rohn, Tanja Schwerdtle, Anna Kipp, Fulvio Ursini, Matilde Maiorino, Giovanni Miotto, Elias S.J. Arnér

**Affiliations:** a Division of Biochemistry, Department of Medical Biochemistry and Biophysics, Karolinska Institutet, SE-171 77, Stockholm, Sweden; b Department of Molecular Medicine, University of Padova, Padova, Italy; c University of Potsdam, Institute of Nutritional Science, Department of Food Chemistry, Nuthetal, Germany; d German Federal Institute for Risk Assessment (BfR), Berlin, Germany; e Friedrich Schiller University Jena, Institute of Nutritional Sciences, Molecular Nutritional Physiology, Jena, Germany; f CRIBI Biotechnology Center, University of Padova, Padova, Italy; g Department of Selenoprotein Research, National Institute of Oncology, Budapest, Hungary

**Keywords:** Recombinant selenoprotein, Glutathione peroxidase, GPX1, GPX2, GPX4, Frameshift

## Abstract

Selenoproteins are translated via animal domain-specific elongation machineries that redefine dedicated UGA opal codons from termination of translation to selenocysteine (Sec) insertion, utilizing specific tRNA species and Sec-specific elongation factors. This has made recombinant production of mammalian selenoproteins in *E. coli* technically challenging but recently we developed a methodology that enables such production, using recoding of UAG for Sec in an RF1-deficient host strain. Here we used that approach for production of the human glutathione peroxidases 1, 2 and 4 (GPX1, GPX2 and GPX4), with all these three enzymes being important antioxidant selenoproteins. Among these, GPX4 is the sole embryonically essential enzyme, and is also known to be essential for spermatogenesis as well as protection from cell death through ferroptosis. Enzyme kinetics, ICP-MS and mass spectrometry analyses of the purified recombinant proteins were used to characterize selenoprotein characteristics and their Sec contents. This revealed a unique phenomenon of one-codon skipping, resulting in a lack of a single amino acid at the position corresponding to the selenocysteine (Sec) residue, in about 30% of the recombinant GPX isoenzyme products. We furthermore confirmed the previously described UAG suppression with Lys or Gln as well as a minor suppression with Tyr, together resulting in about 20% Sec contents in the full-length proteins. No additional frameshifts or translational errors were detected. We subsequently found that Sec-containing GPX4 could be further purified over a bromosulfophthalein-column, yielding purified recombinant GPX4 with close to complete Sec contents. This production method for homogenously purified GPX4 should help to further advance the studies of this important selenoprotein.

## 1 Introduction

In biological systems, selenium (Se) exerts its main functions as selenocysteine (Sec), the 21st amino acid and the defining entity of the unique class of proteins named selenoproteins [[1], [2], [3], [4]]. Sec biosynthesis occurs on its cognate tRNA^[Ser]Sec^ in a highly complex process, with co-translational incorporation of Sec at a predefined UGA opal codon requiring the orchestrated action of a series of factors, including a Sec-dedicated elongation factor interacting either directly or indirectly with a so called SECIS (Sec insertion sequence) element in the selenoprotein-encoding mRNA [[1], [2], [3], [4], [5]]. The complicated translation machineries for selenoproteins are furthermore animal domain-specific, with the natural genes encoding any of the 25 human selenoproteins not being compatible with the translation machinery in *E. coli*. This fact makes the direct expression of recombinant human selenoproteins in *E. coli* impossible [1,[6], [7], [8], [9]]. However, previously it was found that by utilizing a bacterial-type SECIS element to by-pass these species barriers, production of mammalian selenoproteins in *E. coli* can be enabled, but typically only when the Sec residue is located close to the C-terminal end of the selenoprotein such as in thioredoxin reductases or Sel-tagged proteins [[7], [8], [9], [10], [11], [12], [13]]. That methodological restriction in recombinant selenoprotein synthesis was recently circumvented using Sec-mediated suppression of UAG instead of UGA, utilizing a complementary mutated bacterial tRNA^Sec^ species together with overexpression of the bacterial SelB elongation factor, in the absence of a SECIS element but in an *E. coli* host strain lacking release factor 1 (RF1) that would otherwise catalyze translational termination [14,15]. This highly artificial bacterial synthesis machinery indeed enables production of Sec-containing recombinant selenoproteins, in combination with Sec-deficient expression products being the result of Lys- and Gln-suppression at the position of the UAG codon [14]. In the present study we further analyzed the products of this selenoprotein production system, and specifically studied the three major human cytosolic glutathione peroxidase (GPX) family members when expressed in recombinant forms.

GPXs are highly efficient enzymes reducing peroxides at the expense of glutathione (GSH) and encompass eight family members in human, five of which are selenoproteins [16]. In the present study, we aimed at producing in recombinant form the three intracellular selenoproteins of this family, namely GPX1, GPX2 and GPX4. Of these, GPX1 is the main cytosolic GPX enzyme, GPX2 is specifically expressed in the intestine and other epithelial cells, and GPX4 is the only GPX family member known to be dedicated at reducing lipid peroxides at the membrane [[17], [18], [19], [20], [21]]. GPX4 is also an essential selenoprotein for mammals, as shown in knockout mouse models [22], which moonlights into a structural component of the maturing sperm [17,[23], [24], [25], [26]], as well as protects cells from ferroptotic cell death [27,28]. Drug targeting of GPX4 to trigger ferroptosis in cancer cells has been proposed as a promising anticancer therapy principle [19,[29], [30], [31]]. We thus reasoned that being able to produce and compare recombinant forms of the different GPX family members should have significant interest in the general field of recombinant selenoprotein production, as well as in relation to the importance of specific GPX enzymes. Our analyses of the hereby produced recombinant selenoproteins revealed unexpected insights with regards to the characteristics of the bacterial selenoprotein production machinery, showing single-codon skipping, as well novel insights with regards to distinct features of different GPX4 variants in binding to BSP Sepharose enabling essentially homogenous purification of this particular selenoprotein.

## 2 Materials and methods

### 2.1 Chemicals, reagents, plasmids and *E. coli* strains

We previously described a method for production of recombinant selenoproteins in *E. coli* using a host strain lacking RF1 and recoding UAG as a Sec codon, utilizing overexpression of the tRNA for Sec with a corresponding mutation compatible with UAG and overexpressing the SelB elongation factor together with the SelA selenocysteine synthase. In that system we produced recombinant human thioredoxin reductase 1 (TrxR1, also named TXNRD1) and GPX1 [14]. Here we aimed to express additional selenoproteins in this system, whereby the open reading frame (ORF) of human glutathione peroxidase 2 (GPX2, GenBank: CAA48394.1, residues 1-190), human glutathione peroxidase 4 (GPX4, GenBank: AAH22071.1, residues 28-197), and human thioredoxin reductase 2 (TrxR2, also named TXNRD2, GenBank: NP_006431.2, residues 37-524), codon optimized for recombinant expression in *E. coli*, were synthesized by Integrated DNA Technologies, Inc. All constructs were made using UAG as the Sec codon and for the TrxR2 construct, a bacterial SECIS element was positioned 11 nucleotides downstream of the UAG codon and outside its entire ORF as described previously for TrxR1 [14]. The different ORF were subcloned into the inhouse pABC2a plasmid (pABC2a-HsGPX4) that generates a fusion protein of the corresponding His6-SUMO-HsGPX2, His6-SUMO-HsGPX4 and His6-SUMO-HsTrxR2, respectively. In this nomenclature, His6 refers to an N-terminal His-tag for IMAC purification, SUMO (small ubiquitin-related modifier) to a 110-residue sequence recognized by SUMO protease ULP1 that hydrolyzes the peptide bond at the C-terminus of the SUMO domain, which results in release of the target selenoproteins GPX2, GPX4 or TrxR2 from their N-terminal fusion partners. These plasmids were subsequently transformed into the RF1 depleted *E.coli* strain C321.ΔA (Addgene Item #48998) [15] for selenoprotein production, as described below.

Human glutathione reductase (GSR, GenBank: AAP88037.1, residues 1-479) was also produced for this study. Briefly, the ORF for GSR was synthesized with codons optimized for *E. coli* expression and subcloned into the in-house developed pD441 plasmid that generates a fusion protein named His6-SUMO-HsGSR, with the plasmid transformed into BL21(DE3) for protein production. The procedure for GSR expression and purification is similar to the selenoproteins production detailed below, except that the culture medium contained 50 μg/ml kanamycin, culture temperature before IPTG induction was 37 °C, and no additional selenite was added.

All sequences of constructs used for recombinant protein production in this study are summarized in Table 1. Chemicals and reagents were typically obtained from Merck/Sigma-Aldrich unless specified otherwise. The phosphatidylcholine hydroperoxide (PC-OOH) substrate was synthesized through an enzymatic oxidation procedure as described earlier [17].

**Table 1:**
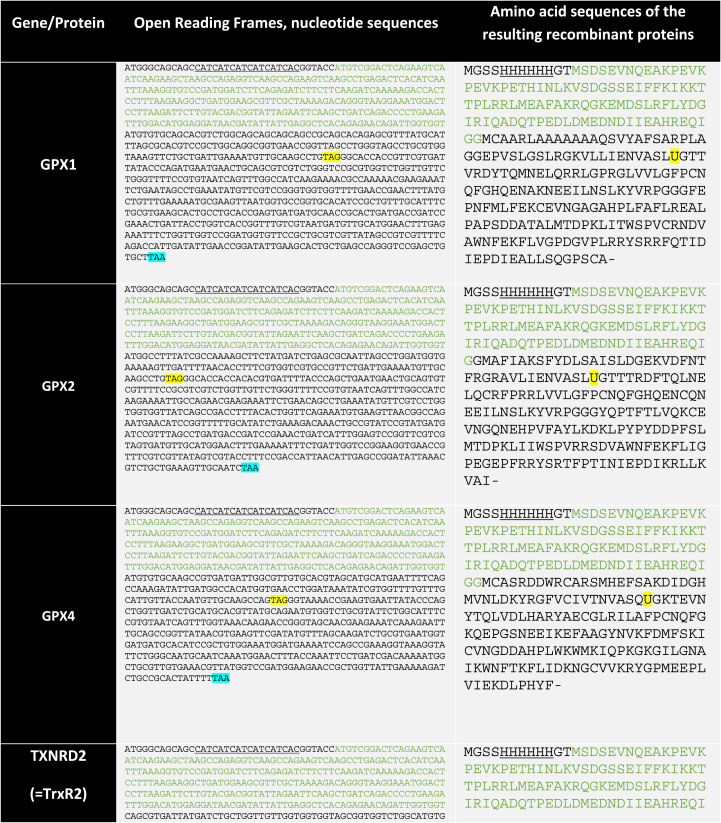

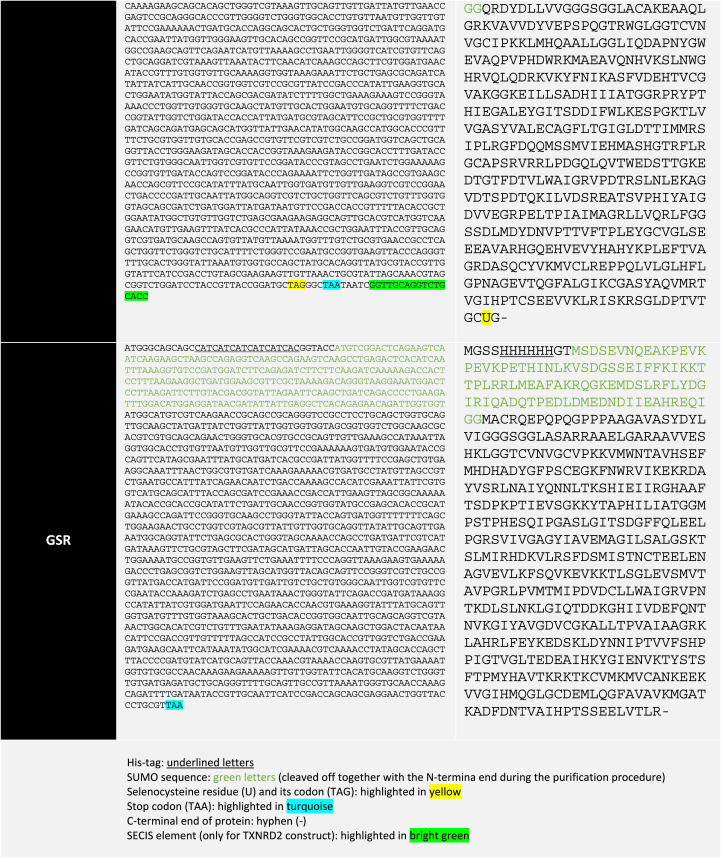
Sequences of the specific plasmid-encoded open reading frames and the resulting recombinant proteins used in this study. All constructs made for this study were synthesized to be codon optimized for *E. coli* expression, as detailed in the Methods section. This table summarizes the nucleotide sequences of the open reading frames and the resulting amino acid sequences of the expressed recombinant proteins, with key features indicated as given in the table footnote.

### 2.2 Production and initial purification of recombinant selenoproteins

The recombinant selenoproteins were first expressed and purified essentially as described previously [14]. Briefly, 40 ml of overnight cultures of transformed bacteria were inoculated into 2 L terrific broth (TB) medium containing 50 μg/ml streptomycin and 50 μg/ml carbenicillin in a 5 L bottle placed on a shaking incubator at 30 °C. At 6 h after inoculation, temperature was lowered to 25 °C and 0.5 mM IPTG together with 5 μM sodium selenite were added to induce selenoprotein expression overnight. The bacteria were subsequently harvested by centrifugation, suspended in IMAC binding buffer (50 mM Tris-HCl, 100 mM NaCl, 10 mM imidazole, pH 7.5) and lysed by sonication. The soluble fraction was recovered by centrifugation and applied onto a HisPrep FF 16/10 column equipped on an ÄKTA explorer FPLC system (Cytiva Life Sciences). The eluted fusion protein was treated with inhouse produced His-tagged ULP1 (1%) and subsequently re-applied onto the HisPrep FF 16/10 column to separate non-tagged target protein from its N-terminal His-tagged fusion partner as well as from the His-tagged ULP1. The target proteins were then concentrated, the buffer was exchanged, and the proteins were stored in −20 °C freezer until analyses. The buffer for stock solutions of GPX preparations was 50 mM Tris-HCl, pH 7.5, with 100 mM NaCl, 5 mM 2- mercaptoethanol, and 20% glycerol, while the buffer for TrxR2 was 50 mM Tris-HCl, pH 7.5, with 2 mM EDTA, and 20% glycerol. The purity of the final selenoprotein was always greater than 95% as assessed by SDS-PAGE.

### 2.3 Enzyme kinetics

GPX activity was initially measured using GSH with either H_2_O_2_ or cumene hydroperoxide (CHP) as substrates, in an assay coupled with glutathione reductase (GSR) and NADPH. Reactions were carried out in either 96-well plates or 1-cm cuvettes with 1 mM GSH, 0.5 mM H_2_O_2_ or CHP, 15 nM human GSR, and 0.2 mM NADPH. The NADPH consumption was monitored by measuring the change in absorbance at wavelength of 340 nm over time.

Kinetic constants were determined as described [17,32]. Briefly, reactions were performed in 0.1 M KH_2_PO_4_/K_2_HPO_4_ pH 7.8 containing 5 mM EDTA, 0.1% (v/v) Triton X-100, 1.6 × 10^−4^ M NADPH and 0.6 IU/ml Glutathione Reductase (GSR; Sigma) with different GSH concentrations (2 × 10^−3^, 3 × 10^−3^, 4 × 10^−3^ M). These reactions were carried out at 25 °C and initiated by addition of the peroxide substrate (2 × 10^−5^ M), where PC-OOH was dissolved in methanol and H_2_O_2_ in water. Absorbance data at 340 nm were collected from progression curves of NADPH oxidation using a Cary UV–Vis multicell Peltier spectrophotometer with the 6220 M^−1^cm^−1^ extinction coefficient used for calculations. All kinetic analyses were carried out from single progression curves of NADPH oxidation at different GSH concentrations with absorbance change over time used to calculate substrate concentrations and enzymatic rates at time intervals of 5 s. The enzyme concentrations used for the kinetics determinations were 6.67 × 10^−8^ M and 3.42 × 10^−8^ M for recombinant GPX4, with PC-OOH and H2O_2_, respectively, while GPX1 was used at 1.79 × 10^−9^ M for both substrates. The rate of the reaction catalysed by GPX4 was corrected for the rate of the reaction with the same substrate, at the same concentrations, but lacking enzyme. No correction was needed with only PC-OOH. Apparent rate constants for GPX4 were calculated by the simplified Dalziel equation:

E/v_0_ = φ_0_ + φ_1_/[ROOH] +φ_2_/[GSH]

where:

φ_0_ is the reciprocal of the turnover number, using 0 for GPX4 as for the other GPXs; φ_1_ and φ_2_ are the Dalziel coefficients, being equivalent to the reciprocal second order rate constant of the peroxidatic and reductive steps of the reaction, respectively.

### 2.4 Se contents determinations

Se contents was determined with an ICP-QQQ-MS mass spectrometer (Agilent, Waldbronn, Germany), essentially as described [33]. In short, microwave-digested samples consisting of 1.5 ml in total were analyzed with 90 μl protein sample, 45 μl (100 μg/l) ^77^Se, 15 μl (100 μg/l) Rh, 1350 μl 20% HNO_3_ and 45 μl isopropanol (3% final concentration). Blanks were made with 1440 μl 20% HNO_3_, 45 μl (100 μg/l) ^77^Se, 15 μl (100 μg/l) Rh. For reference material, ClinChek Control Serum Trace Elements Level 2 and Seronorm Trace Elements Urine L-1 were used.

### 2.5 Synthesis of BSP-Sepharose

First, the functional group was synthesized by allowing 1.5 g of BSP (bromosulfophthalein; Sigma S0252) to react with 1 g GSH in 60 ml sodium bicarbonate buffer (0.1 M, pH 10.2), with the buffer pre-equilibrated at room temperature with nitrogen. Conjugation was then allowed to occur at room temperature for about 24 h in a tightly closed flask under stirring. The reaction product was subsequently precipitated by adding drop by drop of about 800 ml cold acetone (kept at −20 °C). After acetone addition the precipitate was allowed to form for 10 min at room temperature under continuous agitation, whereupon the precipitation mixture was placed at −80 °C for 3–4 h. After this period, the adduct, which was largely attached to the walls of the container in the form of a sticky compound, was filtered (Borosilikat filter 3.3 Robu glass por. 4) and dried in a SpeedVac (ThermoFisher) for elimination of residual acetone. With the BSP-GSH adduct being difficult to remove from the filter, everything was kept tightly closed with parafilm and stored at −80 °C.

Second, the resin was prepared by first adding 300 ml Sepharose 4B (Pharmacia/Sigma-Aldrich) to a filter connected to a water pump, with the resin subsequently washed several times with double distilled water and subsequently resuspended in 300 ml 2 M sodium carbonate (pH 11). Then 25 g of CNBr was dissolved in 8 ml of acetonitrile and added to the resin under stirring, with activation allowed at room temperature for 2 min. The activated gel was then transferred to the filter connected to a vacuum pump and washed with at least 4 L ice-cold 10 mM potassium phosphate buffer (pH 7.5) containing 0.1 M KCl (activation being dangerous and has to be carried out in a hood, using gloves and having concentrated NaOH in which all glassware coming into contact with CNBr has to be put).

Finally, the BSP-GSH adduct was conjugated with the activated Sepharose 4B gel by dissolving the adduct in 10 mM potassium phosphate buffer (pH 7.5) containing 0.1 M KCl and 1 mM EDTA, using the least possible volume to allow the concentration of adduct to be high during the reaction. The mixture of gel and adduct was placed at 4 °C with slow rotation for 48 h. After the conjugation reaction, the gel was filtered and washed intensively with 10 mM potassium phosphate buffer (pH 7.5) containing 0.1 M KCl and 1 mM EDTA for removal of all free adduct, whereupon it was loaded in a column for use in the BSP affinity chromatography.

### 2.6 Purification of GPX enzymes over BSP affinity chromatography

For GPX4 purification, either 2 mg of recombinant GPX4 or native GPX4 purified from human placenta were first diluted 1: 50 in loading buffer (10 mM KH_2_PO_4_/K_2_HPO_4_, pH 7.0 containing 10% glycerol and 5 mM 2-mercaptoethanol) and loaded onto the column at 1 ml/min. Then a three-bed column volume washing with loading buffer followed by elution buffer A (25 mM Tris-HCl, 50 mM KCl pH 7.8 containing 10% glycerol and 5 mM 2-mercaptoethanol). Elution of GPX4 was subsequently accomplished by a stepwise gradient with 48%, 62%, 75% and 100% elution buffer B (25 mM Tris-HCl, 500 mM KCl pH 8.3 containing 10% glycerol and 5 mM 2-mercaptoethanol).

For GPX1 purification, 0.25 mg of recombinant GPX1 was diluted 1: 50 in loading buffer and loaded onto the column at 1 ml/min followed by a three-bed column volume washing with loading buffer (10 mM KH_2_PO_4_/K_2_HPO_4_, pH 6.3 containing 10% glycerol and 5 mM 2-mercaptoethanol). Elution was then accomplished by a stepwise gradient with 25%, 50%, 75% and 100% elution buffer (10 mM KH_2_PO_4_/K_2_HPO_4_, 200 mM KCl pH 7.5 containing 10% glycerol and 5 mM 2-mercaptoethanol).

### 2.7 Mass spectrometric analyses

#### 2.7.1 LC-MS analysis

Samples were analyzed by LC-MS/MS using a 6520 Q-TOF mass spectrometer controlled by Agilent MassHunter software (B.05.00 version), coupled online with a 1200 series HPLC system through a Chip Cube nano-ESI interface (Agilent Technologies, CA, USA). Chromatographic separations of intact proteins were performed on a reverse-phase, 5 μm C8 chip-column (Zorbax C8 - 0.075 mm × 40 mm, Agilent Technologies), integrating a 40 nl capacity trap-column, and a nano-spray emitter.

Peptides from tryptic digestion of GPX4 were separated on a reverse-phase, high resolution 3 μm C18 chip-column (Polaris-HR-3C18 – 0.075 mm × 150 mm, Agilent Technologies), integrating a 360 nl capacity trap-column, and a nano-spray emitter.

#### 2.7.2 Intact protein analyses by LC/MS

GPX samples were diluted to about 10 μg/ml with high ionic strength buffer (100 mM Tris-HCl, pH 7.5, 250 mM NaCl, 2 mM EDTA and 10% glycerol) then, just before LC/MS analysis, reduced in 400 mM DTT for 1hr (ratio sample/3 M DTT, 6/1). The reduction step was introduced since the GPX Sec residue spontaneously shifts to a selenenylamide form (-2H) in non-reducing buffer and, depending on previous sample treatments, to remove 2-mercapto-ethanol adducts from Cys and Sec [34].

Protein amounts, loaded on LC/MS, ranged from 150 to 500 fmol, a switching valve between the pre-column and the analytic column allowed the fast and complete removal of the salts before MS analysis by flushing 4 μl of an aqueous buffer containing 0.1% formic acid and 25% acetonitrile. Samples were resolved at 0.3 μl min^−1^ with a gradient.

Buffer A was aqueous 0.1% formic acid, and Buffer B acetonitrile/methanol - 90/10, 0.1% formic acid. The percentage of B increased from 28% to 44% in 10 min, from 44% to 70% B in 5 min. Re-equilibration was performed by 28% B for 10 min. Eluted proteins were ionized at 1.7 kV, fragmentor set at 250 V, source gas temperature was 325 °C and gas flow rate 4.8 l/min. Acquisition parameters were set at an MS scan rate of 1 spectra/s in the range between 130 and 3200 *m*/*z* in high-resolution mode (R = 20.000).

Data were acquired in profile mode and analyzed with MassHunter Workstation Software Qualitative Analysis rel. B06 (Agilent Technologies, Santa Clara, CA, USA). Deconvolution of MS signals from whole protein was performed with pMod, an improved version of maximum entropy algorithm, in an *m*/*z* range encompassing at least seven differently charged clusters. The baseline subtraction factor was set to its minimum value, relative height peak filters to 1% of the highest peak, and significance filter to a value greater or equal to 25,00. Target mass range used for deconvolution was 6000 to 60,000 Da. All other parameters were left at their default values.

#### 2.7.3 In-gel digestion of recombinant GPX4

One microliter of the recombinant GPX4 preparation (4 μg/μl) was diluted with nine μl of Laemmli sample buffer in reducing conditions (5% 2-mercaptoethanol) and loaded onto a precast 4–12% SDS-PAGE slab (NuPAGE; Thermo Fisher Scientific). The electrophoretic process (180 V constant) proceeded until bromophenol blue reached the end of the gel. The gel was then stained with SimplyBlue Safe Stain (Invitrogen) and destained with water. The band at about 19 kD was manually excised, cut into small pieces, and treated alternately with several washes of water and acetonitrile. After the last wash with acetonitrile, the gel pieces were dried under vacuum and then treated with 200 μl of 10 mM DTT (Sigma) in 50 mM NH_4_HCO_3_ for 1 h at 56 °C and successively with 200 μl of 55 mM iodoacetamide (IAA; Sigma) in 50 mM NH_4_HCO_3_ for 45 min at room temperature and in the dark. The gel was then repeatedly washed with 200 μl of 50 mM NH_4_HCO_3_ and acetonitrile. After dehydration under vacuum, gel pieces were incubated with 30 μl of sequencing-grade modified trypsin (12.5 ng/μl in 50 mM NH_4_HCO_3_; Promega) overnight at 37 °C. Peptides were extracted from the gel using three changes of 50% acetonitrile/0.1% formic acid. The sample was dried under vacuum and kept at −20 °C until LC-MS/MS analysis.

#### 2.7.4 Tryptic peptide identification by LC-MS/MS

Tryptic peptides were re-suspended with 20 μl of 5% acetonitrile/0.1% formic acid and 2 μl analyzed by LC-MS with a linear gradient from 5% to 50% solvent B in 30 min at a flow rate of 0.3 μl min^−1^. Solvent A was water with 0.1% formic acid, while B was acetonitrile with 0.1% formic acid. Mass spectra were obtained in a data-dependent mode: MS/MS spectra of the four most intense ions were acquired for each MS scan in the 140–1700 Da range. The scan speed was set to 3 MS spectra/s and 3 MS/MS spectra/s. The capillary voltage and the drying gas flow rate were set to 1720 V and 4 l/s. Raw data files were converted into Mascot Generic Format (MGF) files with MassHunter Qualitative Analysis Software (Agilent Technologies). MGF files were analyzed using Mascot Search Engine, server version 2.3 (Matrix Science, London, UK). MS/MS datasets were searched against an ad hoc built database for GPX4 containing all the possible amino acid substitutions for the selenocysteine. Enzyme specificity was set to trypsin with up to 2 missed cleavages; the peptide and the fragment tolerances were set to 6 ppm and 0.06 Da, respectively. Carbamidomethylation on cysteine/selenocysteine was selected as a fixed modification. Oxidation of Met residues, deamidation on arginine/glutamine, and dehydroalanine from selenocysteine were set as variable modifications.

### 2.8 In silico analyses

The crystal structure of human GPX4 (PDB code: 6HN3) was retrieved from the PDB [35]. All water molecules and ligands were removed; protein was prepared by adding hydrogen atoms using standard geometries using Molecular Operating Environment (MOE) [36]. To minimize contacts between hydrogens, the structure was subjected to FF19SB force field minimization until the root mean square deviation of conjugate gradient was <0.1 kcal mol^−1^ Å^−1^. Protein charges were added using the Protonate 3D tool of the MOE.

The different GPX4 variants (wildtype, Sec-to-null, Sec-to-Gln and Sec-to-Lys) were subjected to Molecular Dynamics simulation (MD) using ACEMD [37] with explicit water molecules (n = 9616, TIP3P model). For the parameterization of the system FF19SB force field was used in ACEMD [37]. The equilibration phase of the system was performed with ACEMD [37]. Firstly, a solvent equilibration (1 ns) was obtained applying positional restraints on carbon atoms. Secondly, 10 ns molecular dynamics was performed on the full system. The equilibration phase was performed by using NPT ensemble (isothermal, isobaric), at constant pressure (1 atm, Berendsen method) and temperature (300 K, Langevin thermostat). To obtain the final configuration of the proteins, each molecular system was simulated for 100 ns (NPT, 1 atm, 300 K as explained above).

BSP in the phenolic form (BSPp, pH 7) and in the quinoidal form (BSPq, pH 8.3) was modeled, minimized and charged using the MMFF94x force field of MOE. To identify sites compatible with both the dimension and the chemical characteristics of BSP, a Site Finder (MOE) approach was used, as reported in Ref. [20]. The most promising area identified includes Gly 128, Ile 129, Leu 130, Gly 131, Asn 132, Ala 133, Ile 134, Lys 135, Trp 136, Arg 152, Gly 154, Pro 155, Met 156, GLu 157.

Docking experiments were performed using the MOE Dock program; Triangle Matcher was exploited as the placement method while GBVI/WSA dG as the docking scoring function. The final ligand-protein complexes were obtained after an MD simulation (50 ns) using ACEMD [37] in the conditions described above.

To evaluate the binding affinity of BSP docking poses after MD, X-Score was used [38]. X-Score is an empirical scoring functions which estimate the binding affinity of a given protein-ligand complex, including terms accounting for van der Waals interaction, hydrogen bonding, deformation penalty, and hydrophobic effect. The estimated binding affinity is expressed as a dissociation constant of the protein-ligand complex in negative logarithm (pKd), in which for example a pKd equal to 9 represent a binding affinity in the nanomolar range, while a pKd equal to 6 in the micromolar range. The RMSD (root mean square deviation) reported were calculated using MOE.

## 3 Results

**Fig. 1:**
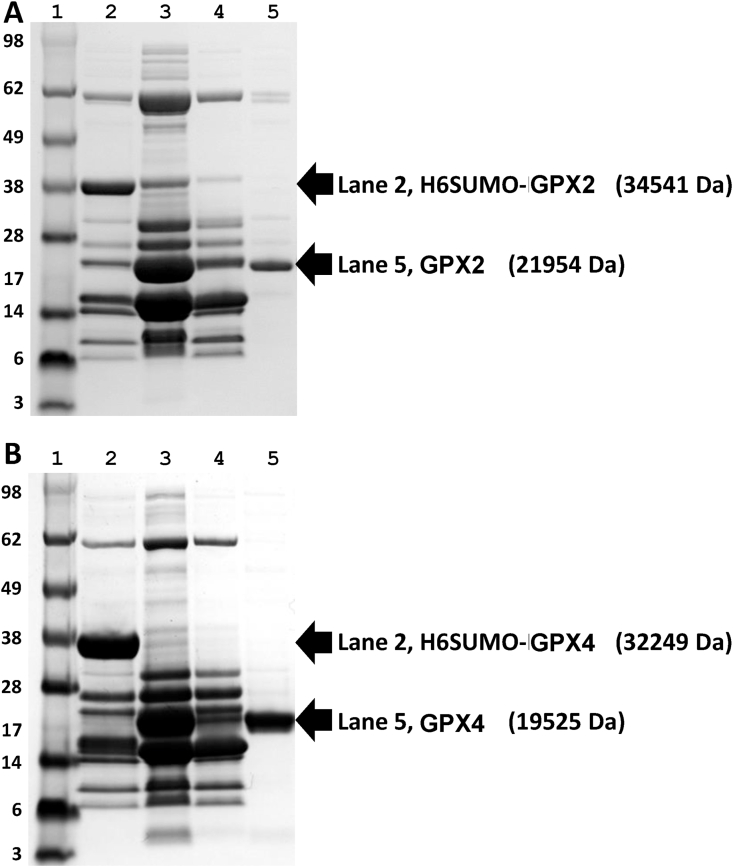
Expression and purification of human GPX2 (A) and GPX4 (B). Lane 1, SeeBlue™ plus2 protein marker (Thermofisher catalog # LC5925). Lane 2, His and SUMO tagged GPX variants (H6SUMO-GPX2 and H6SUMO-GPX4) purified from first IMAC. Lane 3, treatment with His-tagged ULP1 on H6SUMO-GPX variant. Lane 4, proteins bound to the nickel column during second IMAC, including released H6SUMO tag, His-tagged ULP1 and other impurities eluted from first IMAC. Lane 5, flowthrough from second IMAC containing the non-tagged GPX enzymes.

**Fig. 2:**
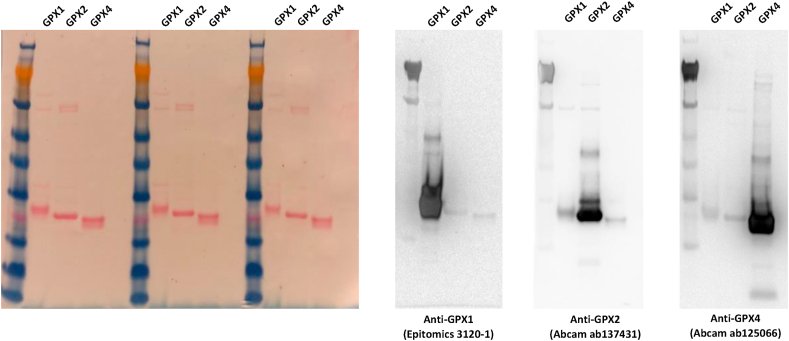
Validating purified recombinant human GPX1, GPX2 and GPX4 using immunoblotting. To the left is shown the Ponceau-S staining of a nitrocellulose membrane after protein transfer from an SDS-PAGE with the purified recombinant GPX isoenzymes ran in triplicates as indicated. The membrane was subsequently cut in three parts, with each part blotted with different primary antibodies as indicated here and in the right part of the figure: Anti-GPX1 (Epitomics catalog # 3120-1), Anti-GPX2 (Abcam catalog # ab137431) and Anti-GPX4 (Abcam catalog # ab125066).

We recently reported how UAG-directed Sec suppression in a RF1-depleted *E. coli* host strain could be used to express and purify recombinant human thioredoxin reductase with close to full Sec contents, and GPX1 with about 20% Sec contents [14]. As we here wished to assess this production system for expression of recombinant human GPX2 and GPX4, which have not before been produced as selenoproteins in *E. coli*, we attempted such production and compared the yields with that for GPX1 as produced earlier. We found that both GPX2 and GPX4 could be expressed and purified to high apparent purity on SDS-PAGE gels (Fig. 1). Total yields of the purified selenoproteins after the final purification steps were typically about 5–10 mg/L bacterial culture. Using immunoblotting with commercial antibodies against GPX1, GPX2 and GPX4 their identities could be further confirmed, and the specificity of the antibodies validated (Fig. 2).

**Fig. 3:**
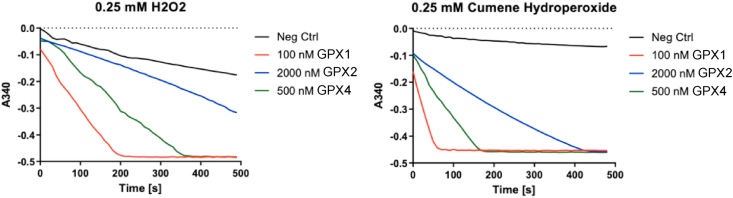
Enzymatic activity of the purified recombinant human GPX1, GPX2 and GPX4 enzymes using either H_2_O_2_ or cumene hydroperoxide as substrates. This figure shows NADPH consumption in assays using either 0.25 mM H_2_O_2_ (left) or 0.25 mM cumene hydroperoxide (right) by following absorbance changes over time at 340 nm in reactions containing glutathione reductase, glutathione and NADPH as outlined in the Methods section, with either only buffer (Neg Ctrl) or the recombinant human GPX1, GPX2 and GPX4 enzymes, at concentrations indicated in the figure.

The preparations of the recombinant human GPX isoenzymes exhibited good enzyme activities and could thereby be used for assessments of enzymatic activity and substrate preference determinations. This was assessed here using the classical GPX substrates H_2_O_2_ and cumene hydroperoxide, using assays coupled with GSH and glutathione reductase and following consumption of NADPH at 340 nm as a result of GSSG turnover. That analysis revealed that all three GPX family members could reduce these substrates, but GPX1 was clearly the most efficient enzyme while GPX2 the least efficient in this direct comparison (Fig. 3). Forthcoming studies could focus on the detailed characterizations of substrate specificities and possible qualitative differences in that regard between individual GPXs.

Although all three GPX enzymes clearly displayed enzymatic activities, albeit with different turnover in the presence of identical substrate concentrations, we reasoned that they may have less than complete Sec contents. As we had previously produced and purified human thioredoxin reductase TrxR1, and based upon activity measurements and mass spectrometric analyses assumed it to have close to full Sec contents while GPX1 was found to have only about 20% Sec contents [14], we here wished to independently assess the Sec contents in the herein purified GPX2 and GPX4 proteins and compare to our previously produced selenoproteins. We also expressed the human thioredoxin reductase isoenzyme TrxR2 and Sec-to-Cys or Sec-to-Ser mutants of GPX1 for further benchmarking. For the analysis of Sec contents in the different protein preparations we used ICP-MS, and determined the sulfur contents as well as the selenium contents in each protein preparation. This enabled a more exact determination of Sec contents in comparison to the theoretical value, when using the number of Cys and Met residues in each protein as internal controls for sulfur contents. These analyses independently confirmed that the previously produced rat TrxR1 with enzymatic activity of 40 U/mg and proposed complete Sec contents [14] indeed has one Sec residue per subunit (here determined as 101.6% of the theoretical value). We also confirmed our previous assessment of about 20% Sec content in recombinant human GPX1 [14], while its Sec-to-Cys or Sec-to-Ser mutants contained no detectable selenium. We also confirmed that the purified recombinant human P190L variant of TrxR1 associated with epilepsy had about half Sec contents, as reported earlier [39], while the recombinant preparations of human TrxR1 and TrxR2 both had about 85% Sec contents. Using this ICP-MS method for Se determination, with normalization to the sulfur contents, we also determined that the recombinant human GPX2 and GPX4 purified herein had 14.0% and 25.4% Sec contents, respectively. These results are summarized in Table 2.

**Table 2:** Determination of Sec contents in recombinant selenoprotein preparations using ICP-MS.

Sample	Sulfur (μM)	Selenium (μM)	Se/S ratio determined by ICP-MS	Cys + Met	Theoretical Sec/(Cys + Met) ratio	Sec contents (%)	Source of sample
Recombinant human GPX enzyme variants							
GPX1	2.80	0.06	0.0203	9	0.1111	18.3	[14]
GPX2	3.73	0.09	0.0234	6	0.1667	14.0	This study
GPX4	8.08	0.16	0.0195	13	0.0769	25.4	This study
GPX1 U49C	24.65	*b.d.*	*b.d.*	10	0	*b.d.*	[14]
GPX1 U49S	18.04	*b.d.*	*b.d.*	9	0	*b.d.*	[14]
*Additional recombinant selenoproteins*							
Rat TrxR1	29.48	1.36	0.0462	22	0.0455	101.6	[14]
Human TrxR1	29.13	1.19	0.0407	21	0.0476	85.5	[14]
Human TrxR2	32.58	1.15	0.0354	24	0.0417	85.1	This study
Human TrxR1 P190L	32.90	0.73	0.0221	21	0.0476	46.5	[39]

*b.d.* = below detection limit.

**Fig. 4:**
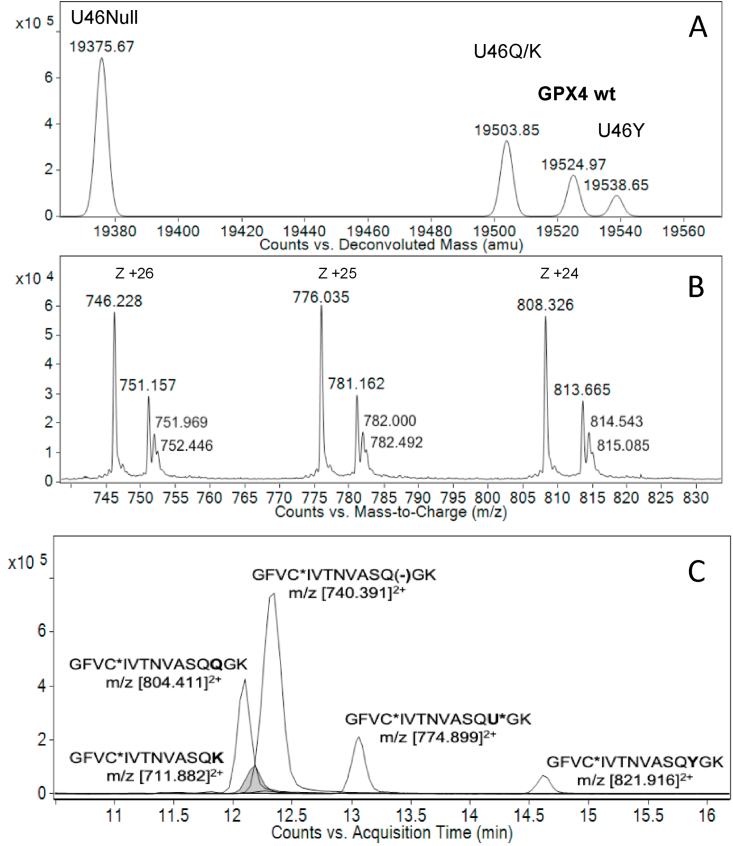
Mass spectrometric analysis of purified recombinant GPX4 species. *A)* The GPX4 species disclosed by mass spectrometric analysis of recombinant GPX4 purified as shown in Fig. 1 are indicated above each peak. The mass values (amu) were obtained by deconvolution of high resolution MS1 spectra as detailed in panel *B*. Panel *C* shows the MS1-extracted ion current over chromatographic retention time of diagnostic tryptic peptides, further supporting the identification of the denoted GPX4 species (see Supplementary Figs. S1 and S2 for further details). (−) indicates the absence of an amino acidic residue, representing the U46Null variant; C* - carbamidomethyl-cysteine; U* - dehydroalanine derivative of Sec.

In our previous study we also found that the production of human GPX1 yielded UAG suppression not only with Sec, but also with Lys and Gln, thereby explaining why the purified protein did not have full Sec contents [14]. In the present study, we determined to analyze such phenomena of non-Sec mediated suppression in further detail, using the newly purified recombinant human GPX4 preparation. We thus asked whether the same phenomenon of Lys or Gln suppression at the UAG codon could fully explain why it only had about 25% Sec contents. It should in this context also be noted that Lys and Gln have very similar mass (35.5 mDa difference) making quantifications of the two separate protein forms difficult in direct mass spectrometry, but the two residues were previously found to be present at about equal amounts in recombinant GPX1 as based upon the profile of protease digest analyses [14]. Using mass spectrometric approaches, we here found that also the recombinant human GPX4 preparation contained Lys or Gln in addition to Sec, at the position corresponding to the UAG codon, as well as a small amount of Tyr suppression. Interestingly, we furthermore detected a “Sec-to-null” variant, indicative of a +3 frameshift event with one-codon skipping during the bacterial translation of the protein (Fig. 4).

The unusual one-codon skipping event was earlier reported when the RF1-deficient *E. coli* strain was used for UAG-directed incorporation of phosphoserine into an overexpressed recombinant protein, and it is likely to be an unusual artifact when using UAG-directed suppression in this particular host strain [40]. This phenomenon of frameshifting should be another factor contributing to the lower specific activity of the purified recombinant GPX4 enzyme since Sec is required in the active site for catalytic activity. We therefore next wished to evaluate whether we could further enrich only the Sec-containing GPX4 species. We reasoned that the Sec-to-null variant is likely to have a distorted three-dimensional structure and thus altered overall properties compared to the wildtype enzyme, noting that when a single amino acid residue is missing from a polypeptide chain the structure of the protein will change, albeit in an unpredictable manner [41]. We also wished to ask whether we could find some purification method that could remove the genuine Sec-containing GPX4 selenoprotein from the Sec-to-Lys, Sec-to-Gln and Sec-to-Tyr variants present from the first purification. Attempting to achieve such enrichments of the Sec-containing GPX4 species we tried a second step of purification over BSP Sepharose.

**Fig. 5:**
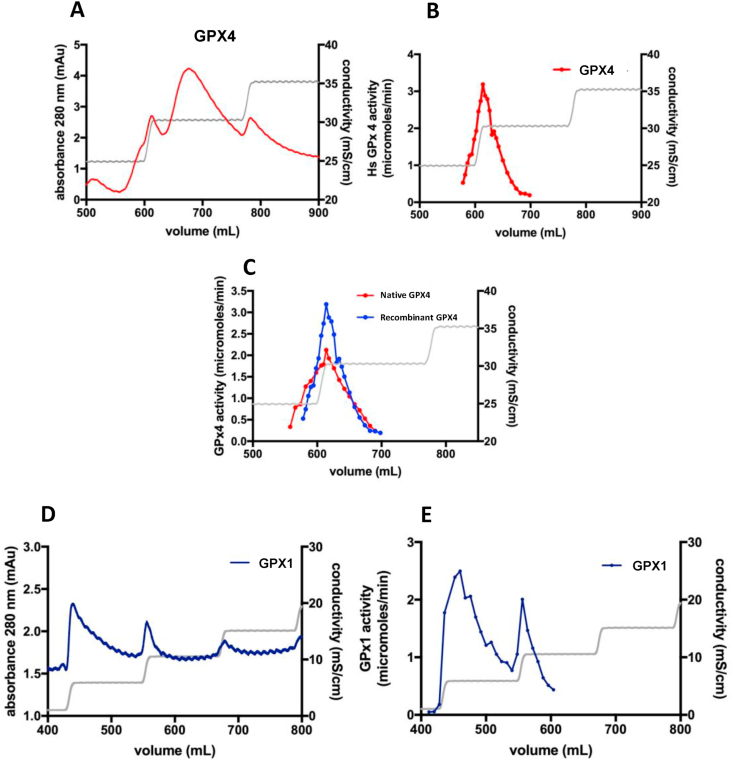
Purification of GPX isoforms over BSP Sepharose. *A)* Chromatogram for recombinant GPX4, purified as shown in Fig. 1 and analyzed using mass spectrometry as in Fig. 4, over BSP Sepharose, showing three peaks of absorbance at 280 nm. *B)* GPX4 activity profile as measured in fractions of the chromatogram shown in *A)*. *C)* Chromatography over BSP Sepharose with GPX4 activity measurements in selected fractions, using as start material either native GPX4 purified from human placenta (red) or recombinant GPX4 (blue). *D)* Chromatogram over BSP Sepharose for recombinant GPX1, purified as shown in Fig. 1, showing limited retention of the protein in the column. *E)* GPX1 activity measurements in fractions of the chromatography shown in *D)*. (For interpretation of the references to colour in this figure legend, the reader is referred to the Web version of this article.)

GPX4 purification over BSP Sepharose was published about forty years ago [42], with this column originally having been developed for purification of GST isoenzymes [43]. However, with elution buffers and schemes later used it was shown that GPX4, and not GST isoenzymes, were purified from crude liver using this column, thus showing good specificity in the purification protocol for GPX4 [42]. Here, using that purification step with the previously purified recombinant GPX4 as starting material, the overall absorbance profile at 280 nm showed three peaks (Fig. 5A), with the enzymatically active GPX4 species eluted at 62% of buffer B corresponding to an increase in conductivity from 25 to 30 mS/cm (Fig. 5B). Importantly, native GPX4 purified from human placenta behaved exactly as the recombinant enzymatically active form of GPX4 (Fig. 5C). We were not able to enrich enzymatically active recombinant GPX1 as efficiently with this column, showing low affinity to the column (Fig. 5D) and activity was spread out in many fractions (Fig. 5E). However, the BSP purification step still increased the specific activity of GPX1 about 3.7-fold, while the specific activity of GPX4 increased more than 18-fold with this additional purification step (Table 3).

**Table 3:** Specific activities of recombinant human GPX1 and GPX4 preparations before and after BSP Sepharose purification. Specific activities were determined with either H_2_O_2_ or PC-OOH as substrates for GPX1 or GPX4, respectively, as described in further detail in the Methods section. The glutathione concentration was 2.5 mM and the total activity recovery over the BSP Sepharose purification step was 85–95% for both enzymes.

Enzyme	Substrate	Specific activity (μmol/min/mg)	
		Before BSP Sepharose	After BSP Sepharose
Recombinant GPX1	H_2_O_2_	20.6	76.6
Recombinant GPX4	PC-OOH	9.20	169.7

Kinetic studies on recombinant BSP-purified GPX1 and GPX4 confirmed their behavior as Se-dependent GPXs. The Linewear-Burk plots obtained with different GSH concentrations produced parallel regression lines, which is consistent with the ping-pong mechanism of these enzymes; moreover, the application of the simplified Dalziel equation showed that φ_0_, the reciprocal of the turnover number, was 0 for both enzymes, as expected where there is no saturation in the presence of increasing concentrations of substrate, as is the case for selenoprotein GPXs. The kinetic parameters φ_1_ and φ_2_ for ROOH and H_2_O_2_ with GSH, respectively, yielding the reciprocal second order rate constant of the peroxidatic and reductive steps of the reaction (Table 4), were comparable to those previously reported for native enzymes [32,44].

**Table 4:** Kinetic constants of recombinant human GPX preparations. Kinetic parameters were determined with either H_2_O_2_ or PCOOH as substrates as described in detail in the Methods section.

Substrate	Recombinant GPX	k_1_ (M^−1^s^−1^)	k_2_ (M^−1^s^−1^)
H_2_O_2_	BSP-purified GPX1	1.36 × 10^6^	1.73 × 10^4^
H_2_O_2_	BSP-purified GPX4	9.55 × 10^4^	4.01 × 10^3^
PCOOH	BSP-purified GPX4	5.28 × 10^6^	2.86 × 10^3^

**Fig. 6:**
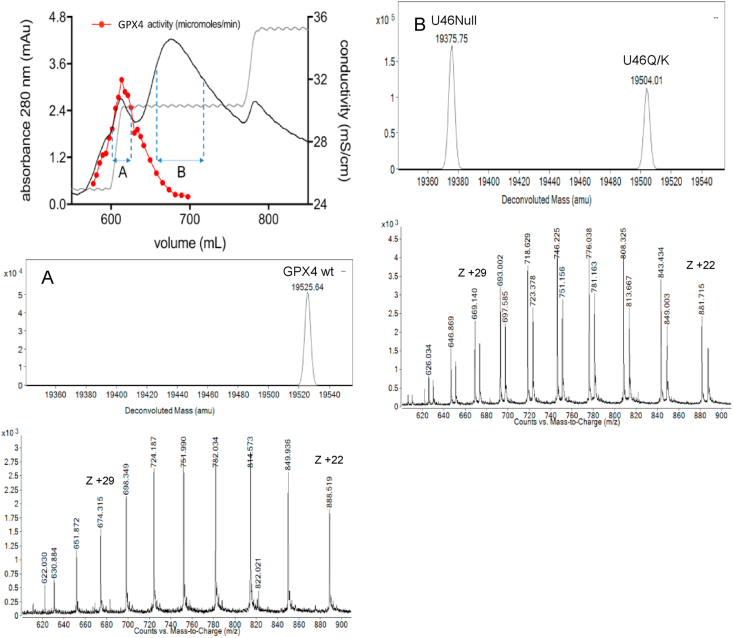
Mass spectrometry analyses of GPX isoforms purified over BSP Sepharose. The top panel indicates the profile of A280 absorbance and GPX4 activity when purified over BSP Sepharose, as shown in Fig. 5A and B, with the eluted proteins in collected fractions further analyzed using mass spectrometry as indicated in A and B. In *A)* the mass-spectrometry spectra and deconvolution of GPX4 species identified in fractions A of the top panel are shown. In *B)* the corresponding analyses of the B fractions are shown.

We next characterized the recombinant BSP-purified GPX4 preparation further using mass spectrometry. These analyses of the different GPX4 species revealed that the first peak from the BSP Sepharose purification, showing enzymatic activity, was indeed almost entirely constituted by Sec-containing GPX4 (Fig. 6A), while the following peaks mainly contained the Sec-to-null and Sec-to-Gln or Sec-to-Lys variants (Fig. 6B). Recovery of the total GPX4 activity applied to the column in the fractions corresponding to the first peak was reproducibly about 75–80% of the initial activity. These results thus revealed that the Sec-containing GPX4 protein could indeed be removed from the other species derived from the recombinant GPX4 production, thus resulting in purified enzyme having essentially complete Sec contents (Fig. 6A).

**Fig. 7:**
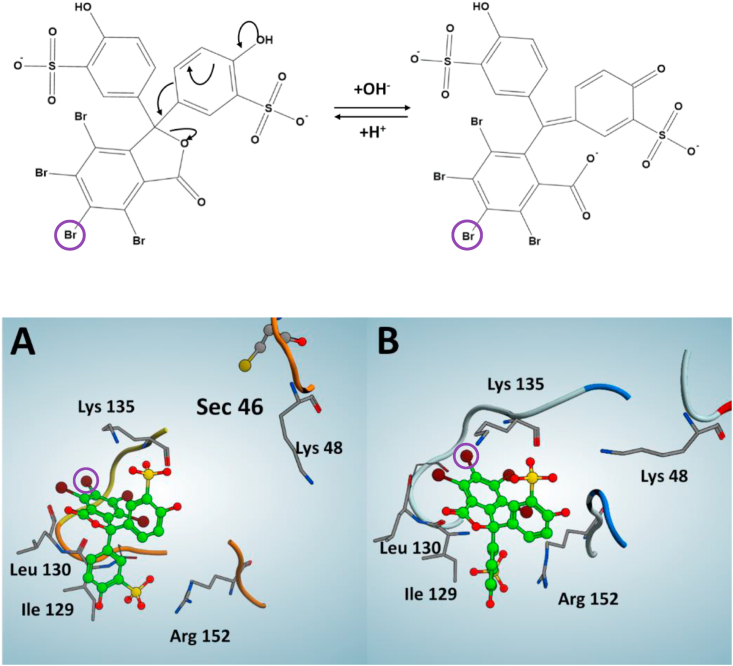
Modeling of GPX isoforms binding to BSP. The top panel indicates the pH dependence in formation of phenolic and quinoidal forms of BSP, with the bromine atom corresponding to the position of the GSH linker in the BSP Sepharose [43] indicated with a purple circle. In *A)* the molecular docking result for wildtype Sec-containing GPX4 interacting with the phenolic form of BSP is shown, with the most important interactions highlighted. The bromine atom showing the position of the GSH linker in the BSP Sepharose is circled as in the top panel for ease of identification. In *B)* the model of the corresponding interaction of BSP with the Sec-to-null variant of GPX4 is shown.

It was interesting to us that Sec-containing GPX4 showed lower affinity to the BSP column than the other Sec-deficient forms of the enzyme, and that GPX1 could not be as efficiently purified on the column (Fig. 5). This suggested to us that the GSH moiety in the column may not necessarily be involved in the binding, and that instead GPX4 specifically interacts with the bromosulfophthalein moiety. This notion was also strengthened by the fact that BSP was previously found to be an efficient reversible inhibitor of GPX4 activity [42]. Attempting to better understand the potential interactions of BSP with GPX4, we next studied this interaction using in silico predictions. In these, we considered both the phenolic and quinoidal forms of BSP (Fig. 7, top panel), which we attempted to dock with the crystal structure of human selenoprotein GPX4 as recently published (PDB code: 6HN3). A site finder approach was used to reveal possible binding areas and the only compatible docking site coincided with an area previously found to be involved in interactions of GPX4 with the polar head of membrane phospholipids [20]. The molecular docking approach suggested a preponderant phenolic conformation of BSP with an incidence of 68%, with respect to the total conformations sampled, and a maximum deviation calculated as RMSD of 1.7 Å. The same computational procedure did not detect any preponderantly sampled conformation for the quinoidal form. The in silico pKd calculated with X-Score for the best ranked conformation of BSP revealed a difference of almost three orders of magnitude between phenolic and quinoidal forms with X-Scores of 7.21 and 4.54, respectively. The result obtained from the docking procedure and calculations of the affinity of the two forms of BSP for GPX4 can elegantly explain why GPX4 could be eluted from the column at pH 8–8.3, since, in this condition, BSP is present in its quinoidal form and therefore shows little affinity for GPX4. The binding site of GPX4 for the phenolic form of BSP is here represented by a surface cavity in which BSP supports the phtalein moiety parallel to a small hydrophobic loop with a Gly-Ile-Leu-Gly sequence, establishing hydrophobic interactions with Ile129 and Leu130 (Fig. 7A). The carbonyl group of the phthalein in BSP when docked with the enzyme is water exposed, while the orientation of the bromine atom corresponding to the position of the GSH linker as present in the purification column is directed away from the enzyme-BSP interaction space. Moreover, the two sulfonate groups of BSP are here modeled to interact electrostatically with the two basic residues Lys135 and Arg152 of GPX4, at 2.73 Å and 2.91 Å distance, respectively (Fig. 7A). This modelling suggests that Sec46 is not directly involved in the BSP Sepharose binding, and that the Sec-to-null variant would thus be able to establish a similar interaction with the column. However, when Sec46 is missing Lys48 can also interact electrostatically with one of the sulfonate groups at 2.90 Å distance (Fig. 7B). This translates into a gain of an order of magnitude of pKd in favor of the BSP-GPX4Sec46-to-null complex (X-Score: 8.5), which is in accordance with the fact that Sec-to-null variant was better retained in the column during purification (Fig. 6). The modeling of the Sec-to-Gln and Sec-to-Lys variants of GPX4 showed a behavior very similar to that of the Sec-to-null variant, however in this case Gln46 and Lys46 could perform a direct electrostatic interaction with one of the sulfonate groups of BSP at 3.01 and 2.95 Å, respectively, also resulting in a significant increase in pKd (X-Score: 8.1 and 8.3, respectively).

These modeling results are thereby fully compatible with the chromatographic profiles of the recombinant protein variants and can explain how it was possible to enrich the Sec-containing GPX4 protein to almost complete purity using the BSP Sepharose column.

## 4 Discussion

In this study we described the results of high-yield expression of human GPX1, GPX2 and GPX4 as recombinant selenoproteins in *E. coli*. Phenomena of an unusual +3 frameshift, resulting in Sec-to-null variants of the proteins, in addition to Sec-to-Gln, Sec-to-Lys and Sec-to-Tyr variants, explain why the purified recombinant proteins have less than 100% Sec contents. For GPX4, we were able to fully enrich the Sec-containing selenoprotein from the other species of the protein using a final BSP Sepharose purification step, and we provided an in silico model explaining the principle for BSP binding of GPX4. We hope that the expression and purification schemes as described in this study can serve as useful methods for enabling forthcoming studies of GPXs, and likely also of many additional selenoproteins.

The phenomenon of a single-codon skipping indicative of a +3 frameshift, resulting in the Sec-to-null variant of the protein that we discovered herein, is interesting. However, we suppose that this is mainly a rather non-natural phenomenon, explained by the features of the engineered *E. coli* host strain lacking release factor RF1 in combination with the tailored production system for recombinant selenoproteins including a non-natural tRNA for Sec with an anticodon for UAG. It should be noted that with this system we had similar results as others studying phosphoserine incorporation at UAG [40], but did not find any evidence of the very extensive frame shifting and base pair skipping phenomena reported for translation in this host strain using a non-overexpressing system lacking a UAG-compatible tRNA species [45].

Although our recombinant selenoprotein production and purification scheme as described herein should be applicable for use with many different selenoproteins, the further enrichment of the Sec-containing GPX4 variant over BSP Sepharose is likely to be a rather specific procedure for the GPX4 isoenzyme. Our in silico modeling helps to explain this purification, with BSP proposed to bind GPX4 in a pH dependent manner at a site not directly involving the Sec residue, but nonetheless explaining how the Sec-to-null and Sec-to-Gln variants could bind the BSP Sepharose stronger than the native form of the enzyme. The possibility of amply producing and purifying large quantities of Sec-containing GPX4 should hopefully come to use for further studies of this important selenoprotein, which is essential for mammals [22,28], protects cells against ferroptosis [18,19,29,31], has been identified as a promising anticancer drug target [29], and with the mitochondrial and nuclear isoforms moonlighting into structural proteins of crucial roles in sperm function and fertilization [[24], [25], [26],^46^,^47^]. It should in this context also be noted that recombinant human GPX4 was recently produced in a mammalian expression system using HEK cells, and purified in such yields and purities so that its crystal structure could be determined [48]. To compare that production and purification scheme for GPX4 with the procedures presented herein should be the topic for future studies, as should further in-depth characterizations of GPX4 and other recombinant selenoproteins with regards to biophysical characteristics and enzymatic properties.

With production and purification of different recombinant selenoproteins now becoming more feasible, as herein illustrated with production of recombinant human GPX1, GPX2 and GPX4, we hope that this rather recently developed methodology shall help to further advance different facets in the many fields of selenoprotein studies.

## Declaration of competing interest

QC and ESJA have shares in Selenozyme AB that is a company selling recombinant selenoproteins such as those described in this study.
