# Supplementary material for: Production and purification of homogenous recombinant human selenoproteins reveals a unique codon skipping event in E. coli and GPX4-specific affinity to bromosulfophthalein
Source: Redox Biol. 2021 Jul 17;46:102070. doi: 10.1016/j.redox.2021.102070 (PMC8326192; doi:10.1016/j.redox.2021.102070)
Supplement: Multimedia component 1 [file mmc1.pdf]

#	b	Seq.	y	#
1	58.0287	G		15
2	**205.0972**	F	1491.7624	14
3	**304.1656**	V	1344.6940	13
4	**464.1962**	C	***1245.6256***	12
5	**577.2803**	I	***1085.5949***	11
6	**676.3487**	V	***972.5109***	10
7	**777.3964**	T	***873.4425***	9
8	891.4393	N	***772.3948***	8
9	**990.5077**	V	***658.3519***	7
10	**1061.5448**	A	***559.2834***	6
11	1148.5769	S	***488.2463***	5
12	**1276.6354**	Q	***401.2143***	4
13	**1345.6569**	U*	***273.1557***	3
14	1402.6784	G	***204.1343***	2
15		K	***147.1128***	1

MS/MS Fragmentation of GFVCIVTNVASQ**U***GK (**GPx4 wt**)
Found in GPX4_HUMAN from (774.899, 2+)
Monoisotopic mass of neutral peptide
Mr(calc): 1547.7766
Fixed modifications: Carbamidomethyl (C)
Variable modifications: U13 : **Secys->Dha (U)**
Matches (**Bold Red**); Ions Score: 99 Expect: 8.7e-10

#	b	Seq.	y	#
1	58.0287	G		14
2	**205.0972**	F	1422.7410	13
3	**304.1656**	V	**1275.6725**	12
4	**464.1962**	C	**1176.6041**	11
5	**577.2803**	I	**1016.5735**	10
6	**676.3487**	V	**903.4894**	9
7	**777.3964**	T	**804.4210**	8
8	**891.4393**	N	**703.3733**	7
9	**990.5077**	V	**589.3304**	6
10	**1061.5448**	A	**490.2620**	5
11	1148.5769	S	**419.2249**	4
12	1276.6354	Q	**332.1928**	3
13	**1333.6569**	G	**204.1343**	2
14		K	**147.1128**	1

MS/MS Fragmentation of GFVCIVTNVASQ(-)GK (**U46Null**)
Found in GPX4_HUMAN from (740.391, 2+)
Monoisotopic mass of neutral peptide
Mr(calc): 1478.7552
Fixed modifications: Carbamidomethyl (C); **Secys->Null**
Variable modifications:
Matches (**Bold Red**); Ions Score: 79 Expect: 6.8e-07

#	b	Seq.	y	#
1	58.0287	G		15
2	***205.0972***	F	1585.8043	14
3	***304.1656***	V	1438.7359	13
4	***464.1962***	C	***1339.6675***	12
5	***577.2803***	I	***1179.6368***	11
6	***676.3487***	V	***1066.5527***	10
7	***777.3964***	T	***967.4843***	9
8	891.4393	N	***866.4367***	8
9	***990.5077***	V	752.3937	7
10	***1061.5448***	A	***653.3253***	6
11	1148.5769	S	582.2882	5
12	1276.6354	Q	495.2562	4
13	***1439.6988***	Y	***367.1976***	3
14	1496.7202	G	***204.1343***	2
15		K	***147.1128***	1

MS/MS Fragmentation of GFVCIVTNVASQ**Y**GK (**U46Y**)
Found in GPX4_HUMAN from (821.916, 2+)
Monoisotopic mass of neutral peptide
Mr(calc): 1641.8185
Fixed modifications: Carbamidomethyl (C)
Variable modifications: U13 : **Secys->Y (U)**
Matches (**Bold Red**); Ions Score: 79 Expect: 6.8e-07

Figure 1- Supplement –MS/MS characterization of recombinant human GPX4 species purified from *E. coli.*
The figure summarizes the fragmentation patterns and distinct features of the key recombinant GPX4-derived tryptic peptides shown in Figure 4C of the main paper.

#	b	Seq.	y	#
1	58.0287	G		15
2	**205.0972**	F	1550.7995	14
3	**304.1656**	V	***1403.7311***	13
4	**464.1962**	C	***1304.6627***	12
5	**577.2803**	I	***1144.6321***	11
6	**676.3487**	V	***1031.5480***	10
7	**777.3964**	T	***932.4796***	9
8	891.4393	N	***831.4319***	8
9	**990.5077**	V	***717.3890***	7
10	**1061.5448**	A	***618.3206***	6
11	**1148.5769**	S	***547.2834***	5
12	**1276.6354**	Q	***460.2514***	4
13	1404.6940	Q	***332.1928***	3
14	1461.7155	G	***204.1343***	2
15		K	***147.1128***	1

MS/MS Fragmentation of GFVCIVTNVASQ**Q**GK (**U46Q**)
Found in GPX4_HUMAN from (804.411, 2+)
Monoisotopic mass of neutral peptide
Mr(calc): 1606.8138
Fixed modifications: Carbamidomethyl (C)
Variable modifications: U13 : **Secys->Q** (U)
Matches (**Bold Red**); Ions Score: 109 Expect: 2.1e-10

#	b	Seq.	y	#
1		G		13
2	***205.0971***	F		12
3	***304.1656***	V	1218.6511	11
4	***464.1962***	C	1119.5827	10
5	***577.2803***	I	***959.5520***	9
6	***676.3487***	V	***846.4680***	8
7	***777.3964***	T	***747.3995***	7
8	891.4393	N	***646.3519***	6
9	***990.5077***	V	532.3089	5
10	***1061.5448***	A	***433.2405***	4
11	1148.5768	S	***362.2034***	3
12	1276.6354	Q	***275.1714***	2
13		K	***147.1128***	1

MS/MS Fragmentation of GFVCIVTNVASQ**K** (**U46K**)
Found in GPX4_HUMAN from (711.882, 2+)
Monoisotopic mass of neutral peptide
Mr(calc): 1421.7337
Fixed modifications: Carbamidomethyl (C)
Variable modifications: U13 : Secys->K (U)
Matches (**Bold Red**); Ions Score: 48 Expect: 4.8e-05

Figure 1- Supplement, cont'd - Characterization of recombinant human GPX4 forms purified from *E. coli.* The figure summarizes the fragmentation patterns and distinct features of the key recombinant GPX4-derived tryptic peptides shown in Figure 4C of the main paper.

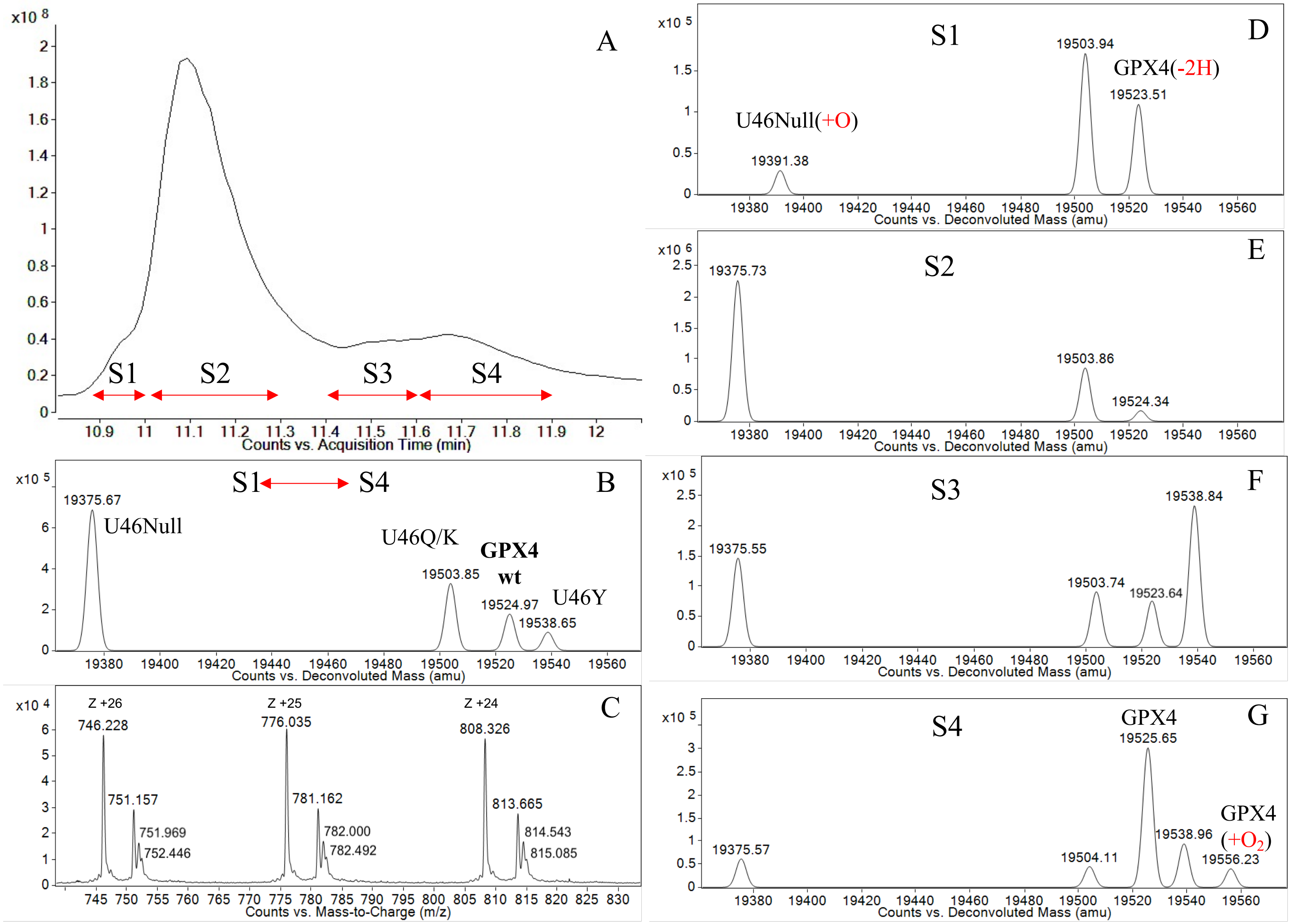

Supplementary Figure 2- RP-HPLC-Mass-Spectrometry characterization of recombinant human GPX4 purified from *E. coli* *A)* RP-HPLC/MS total ion current (TIC) trace. *B)* GPX4 species disclosed by MS, with mass values (amu) obtained by deconvolution of high resolution MS1 spectra as detailed in panel *C*. Panels *D-G* show the GPX4 species identified as in panel *B*, but specifically in the chromatography regions designated S1,S2,S3 and S4 in panel *A*, respectively.
